# A continuous cuffless blood pressure measurement from optimal PPG characteristic features using machine learning algorithms

**DOI:** 10.1016/j.heliyon.2024.e27779

**Published:** 2024-03-12

**Authors:** Araf Nishan, S. M. Taslim Uddin Raju, Md Imran Hossain, Safin Ahmed Dipto, S. M. Tanvir Uddin, Asif Sijan, Md Abu Shahid Chowdhury, Ashfaq Ahmad, Md Mahamudul Hasan Khan

**Affiliations:** a Department of Computer Science and Engineering, Khulna University of Engineering & Technology, Khulna - 9203, Bangladesh; b Department of Software Engineering, American International University, Dhaka, Bangladesh; c Department of Electrical and Electronic Engineering, Dhaka University of Engineering & Technology, Gazipur, Bangladesh; d Department of Biomedical Engineering, Khulna University of Engineering & Technology, Khulna - 9203, Bangladesh

**Keywords:** Continuous blood pressure (BP), Photoplethysmogram (PPG), Feature extraction, Feature selection, Nonlinear regression models

## Abstract

**Background and objective:**

Hypertension is a potentially dangerous health condition that can be detected by measuring blood pressure (BP). Blood pressure monitoring and measurement are essential for preventing and treating cardiovascular diseases. Cuff-based devices, on the other hand, are uncomfortable and prevent continuous BP measurement.

**Methods:**

In this study, a new non-invasive and cuff-less method for estimating Systolic Blood Pressure (SBP), Mean Arterial Pressure (MAP), and Diastolic Blood Pressure (DBP) has been proposed using characteristic features of photoplethysmogram (PPG) signals and nonlinear regression algorithms. PPG signals were collected from 219 participants, which were then subjected to preprocessing and feature extraction steps. Analyzing PPG and its derivative signals, a total of 46 time, frequency, and time-frequency domain features were extracted. In addition, the age and gender of each subject were also included as features. Further, correlation-based feature selection (CFS) and Relief F feature selection (ReliefF) techniques were used to select the relevant features and reduce the possibility of over-fitting the models. Finally, support vector regression (SVR), K-nearest neighbour regression (KNR), decision tree regression (DTR), and random forest regression (RFR) were established to develop the BP estimation model. Regression models were trained and evaluated on all features as well as selected features. The best regression models for SBP, MAP, and DBP estimations were selected separately.

**Results:**

The SVR model, along with the ReliefF-based feature selection algorithm, outperforms other algorithms in estimating the SBP, MAP, and DBP with the mean absolute error of 2.49, 1.62 and 1.43 mmHg, respectively. The proposed method meets the Advancement of Medical Instrumentation standard for BP estimations. Based on the British Hypertension Society standard, the results also fall within Grade A for SBP, MAP, and DBP.

**Conclusion:**

The findings show that the method can be used to estimate blood pressure non-invasively, without using a cuff or calibration, and only by utilizing the PPG signal characteristic features.

## Nomenclature

AAMI: American Association for the Advancement of Medical Instrumentation
*ANN*: Artificial Neural Network
*BHS*: British Hypertension Society
BiLSTM−At: Bidirectional Long Short-term Memory -Attention Neural Network
*BP*: Blood Pressure
CART: Classification And Regression Tree
*CFS*: Correlation-based Feature Selection
*CVD*: Cardiovascular Diseases
*DBP*: Diastolic Blood Pressure
*DWT*: Discrete Wavelet Transform
*FFT*: Fast Fourier Transform
*FIR*: Finite Impulse Response
*GPR*: Gaussian Process Regression
*GRU*: Gated Recurrent Unit
*KNN*: K-Nearest Neighbor
*LR*: Linear Regression
MAE: Mean Absolute Error
*MAP*: Mean Arterial Pressure
*ME*: Mean Difference
*ML*: Machine Learning
*MSE*: Mean Square Error
*PCA*: Principal Component Analysis
*PPG*: Photoplethysmograph
PPGB: Best Single One PPG Cycle
PPGC: Each PPG Cycle
PPGS: Continuous PPG Cycle
*PPT*: Pulse Transit Time
*PWV*: Pulse Wave Velocity
R2: Coefficient of Determination
ReliefF: Relief F feature selection
*RF*: Random Forest
RMSE: Root Mean Square Error
*SBP*: Systolic Blood Pressure
*SVM*: Support Vector Machine
*STD*: Standard Deviation
time_frame: Duration of each PPG Cycle

## 1 Introduction

Blood pressure (BP) measures the force exerted by the circulating blood on the walls of blood vessels. The upper and lower limits of this pressure are referred to as systolic blood pressure (SBP) and diastolic blood pressure (DBP), respectively [1]. Another BP, mean arterial pressure (MAP), can be referred to as equation (1).

(1) MAP=(2DBP+SBP)/3

Hypotension and hypertension are referred to as too low and too high BP [2]. Hypotension occurs when the BP falls below 90/60 [3]. It can be described as a physiological state that indicates different diseases, health conditions, and organ disorders, including anemia [4], cardiogenic shock [5], dizziness, and other conditions. Hypertension, on the other hand, is a more severe condition. It can be considered a frequent condition in which the blood's long-term pressure against the artery walls is high enough to cause heart, brain, kidney, and other cardiovascular issues [6]. If the resting blood pressure is over 140/90, it might be classified as hypertension. It is responsible for 7.5 million fatalities per year, 12.8% of the total of all deaths, more significant than any other risk factors [7]. Another alarming situation would be that roughly 46% of persons with hypertension are ignorant of their disease [8], [9]. Therefore, accurate BP measurement can be highly beneficial in preventing and predicting stroke and cardiac abnormalities and in screening for hypertension in intensive care units, postoperative patients, and people who have recently undergone rehabilitation.

Cuff-based BP measurement techniques are the most commonly used procedure for measuring BP in hospitals. However, it causes discomfort and unpleasant feelings to the patients because of the exerted pressure by the cuff [10]. Even the Cuff-based method, known as white-coat hypertension, can influence blood pressure results [11]. Another issue with the cuff-based method is that it takes a while to get the reading of BP, which leaves continuous monitoring to other counterparts. This results in reliance on invasive measurement procedures, which are performed by inserting a cannula needle into the artery. This invasive procedure leaves a patient uncomfortable and can result in infection. Hence, to reliably measure BP at home, at work, and in hospitals, a non-invasive, continuous, and cuffless BP monitoring system is needed.

**Figure 1:**
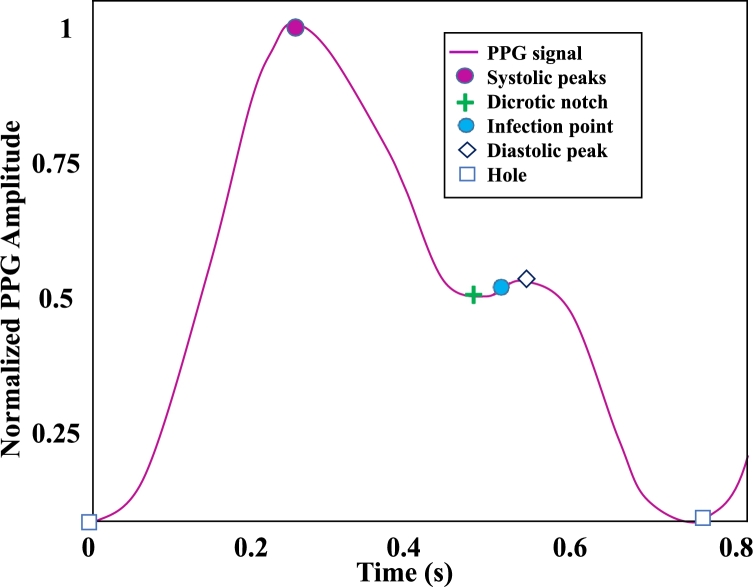
A typical PPG signal with its characteristic features.

Nowadays, wearable sensors can gather critical physiological data for patient monitoring since digital sensors, signal processing, and machine learning algorithms have become more common due to advancements in technology [12], [13]. Previously, non-invasive BP estimation could be done by analysing the PPG pulse wave along with the electrocardiogram wave [14], [15]. In this method, the authors extracted PWV or PTT features from both signals, which is a typical solution. Research has continued to explore techniques for estimating BP using features of the PPG signal only. X. Teng and Y. Zhang in [16] utilised only the PPG signals to measure BP for the first time. Hasanzadeh et al. in [17] proposed a BP estimation method using the PPG signal and its morphological features. PPG is a simple and low-cost optical method for detecting changes in blood volume in the microvascular bed of tissue [18]. PPG data are taken from the outside of the body, including the finger, forehead, earlobe, and toe. These body parts cover veins, arteries, and capillaries for taking records. Infrared light is generally used for blood measurement purposes, penetrating up to 5 millimetres. A precise PPG signal has a wave-like pattern, as shown in Fig. 1. It generally consists of a systolic peak, a diastolic peak, and a dicrotic notch. The comparatively higher peak indicates the systolic peak, and the other indicates the diastolic peak. At the same time, they are separated by the dicrotic notch, which suggests a diastole's beginning and a systole's end [19]. Besides blood pressure measurements, it is also used in a variety of other applications, such as understanding the cardiovascular system [20], measuring heart rate [21], determining oxygen-saturated blood [22], measuring blood glucose levels [18], anemia detection [23] and detecting atrial fibrillation [24]. Researchers have developed ways for determining blood pressure from the PPG signal in recent years using different ML models [12], [25], [26], [27]. An ML-based framework was proposed in [12], where the author implemented the Adaboost model. In [27], the authors used various ML models (LR, RF, SVM, and AdaBoost) to estimate the BP and compare the results with GRU models. RF and SVM models were implemented for non-invasive BP monitoring in [25]. On the other hand, the author in [26] implemented regularised linear regression, SVM, decision tree regression, random forest regression, and Adaboost to estimate the BP. As a result, machine learning algorithms based on PPG signals have been developed to alternate with the traditional BP monitoring approaches. Although various frameworks have been published in recent years, there is always a need for improvement in the precision and robustness of BP estimation.

In this paper, a cuffless and non-invasive BP estimation method has been proposed using the characteristic features of PPG signals and nonlinear regression algorithms. Hence, the PPG signal of 219 subjects was included. After denoising and preprocessing the PPG signals, 46 features are extracted from the PPG signal of each subject. In addition, age and gender information of the subjects have been added as features, and the number of features has been raised to 48. CFS and ReliefF feature selection methods have been applied to select the appropriate feature set. Finally, nonlinear regression algorithms have been developed to estimate BP values. The major contributions of the paper are summarized as follows:

• Extraction of the PPG signals with 2.1 s duration and checking the signal quality.
• Preprocessing the PPG signal using various filtering techniques.
• Development of the peak detection algorithm and extraction of the best PPG cycle from overall PPG waveform.
• Extraction of characteristic features from PPG and its derivative signals.
• Reduction of features using CFS and ReleifF approaches.
• Construction of nonlinear regression algorithms for BP estimation using all features and the selected features.

There are five sections to the manuscript. Section 1 discusses the motivation behind the work and the fundamentals of the PPG signal. Related works in the following field are described in Section 2. The database and methodologies, along with preprocessing procedures, feature extraction, feature selection and different regression techniques are presented in Section 3. Section 4 depicts performance of our proposed method, while Section 5 concludes the paper with future directions.

## 2 Related works

Recently, measuring blood pressure from PPG signals has been an active research area. New proposed architectures, and improvements over existing architectures, carried out by several teams [12], [28] resulted in improvements over earlier studies [29], [30]. In this paper, we pursue the goal of taking the improvements further, improving several previously presented architectures and introducing a novel architecture. A deep neural network based on ResNet was proposed in [31]. The authors used the processed PPG signals and their first and second derivatives as the input of their developed deep learning model (Spectro-temporal ResNet). Hasanzadeh et al. [17] focused on the morphological features of the PPG signal and used the AdaBoostR algorithm as the regression model. Therefore, the proposed method achieved a good correlation between the estimated BP and its actual value.

Mousavi et al. [12] also used an AdaBoostR-based model. The authors denoised their PPG data using FFT and compressed their selected feature vectors (selected over a predefined distance between successive R peaks) using the PCA technique. In [30], [28], authors made use of the CFS algorithm in their feature selection phase. Zhang et al. [30] used LR, SVM and ANN, respectively. The result showed that SVM outperformed all other algorithms. In another study, Chowdhury et al. [28] extracted 65 time-domain and 16 frequency-domain features. The ReliefF feature selection method with the GPR showed promising results for introducing an accurate cuffless BP monitoring system.

Khalid et al. [32] used SVM model as well, along with the regression tree and multiple linear regression. In their study, the regression tree achieved the highest accuracy in estimating BP values. Kauchee et al. [26] on the other hand, achieved better results with random forest and Adaboost. The authors preprocessed the data with wavelet denoising and tested them on different regression models. Dey et al. [33] developed a smartphone application using the lasso regression model. They extracted features from the first four derivatives of the PPG signals and used wavelet smoothing, trend removal, and dynamic peak search in their preprocessing phase. Yi et al. [34] applied KNN to predict BP, compared it with LR, elastic networks, LASSO, and CART, and achieved better accuracy. Similarly, Liu et al. [35] extracted features from PPG signals and it's second derivative, then used support vector regression over them. The authors in [36] also employed a support vector regression model after preprocessing with wavelet transformation. Kurylyak et al. [37] extracted time-scale PPG features proposed by Liu et al. [35] and applied a feed-forward network to estimate BP. The proposed method achieved higher accuracy regarding continuous blood pressure monitoring systems.

Zhang et al. [38] proposed a refined BP prediction strategy utilizing a combination of demographic characteristics (age, gender) and pulse wave morphological features to stratify populations by cardiovascular status before BP estimation. By employing the random forest algorithm, two types of typical CVDs are screened with an impressive accuracy of 92.2%. Additionally, a deep learning model called BiLSTM-At is introduced to estimate the long-term BP trend for different CVD groups. To improve performance and reduce computational complexity, transfer learning techniques are employed for personalized modeling. Kaan Sel et al. [39] proposed a physics-informed neural network model for physiological time series data that can estimate blood pressure using minimal ground truth information. This approach incorporates Taylor's approximation to capture the changing relationships between input and output variables and trains the neural network accordingly.

Although BP estimation from PPG signals has come a long way with recent studies; this work takes it a step further by demonstrating a significant increase in estimating accuracy. In this paper, after collecting PPG characteristic features and selecting relevant features based on the CFS and ReliefF selection methods, selected features were fed into nonlinear regression models.

## 3 Methodology

**Figure 2:**
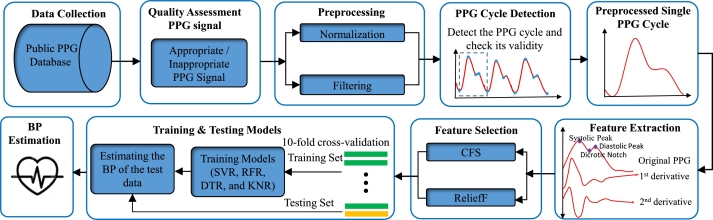
The block diagram of the proposed blood pressure estimation algorithm.

The main steps of our proposed system are as follows: 1) collect the PPG signal from PPG-Database and check the quality assessment, 2) apply the preprocessing algorithms and filter the PPG signals, 3) detect the best PPG cycle and extract the features, 4) select the relevant features using feature selection approaches and 5) finally, establish the machine learning regression algorithms. Fig. 2 depicts overall procedure of the proposed system.

### 3.1 Data description

**Figure 3:**
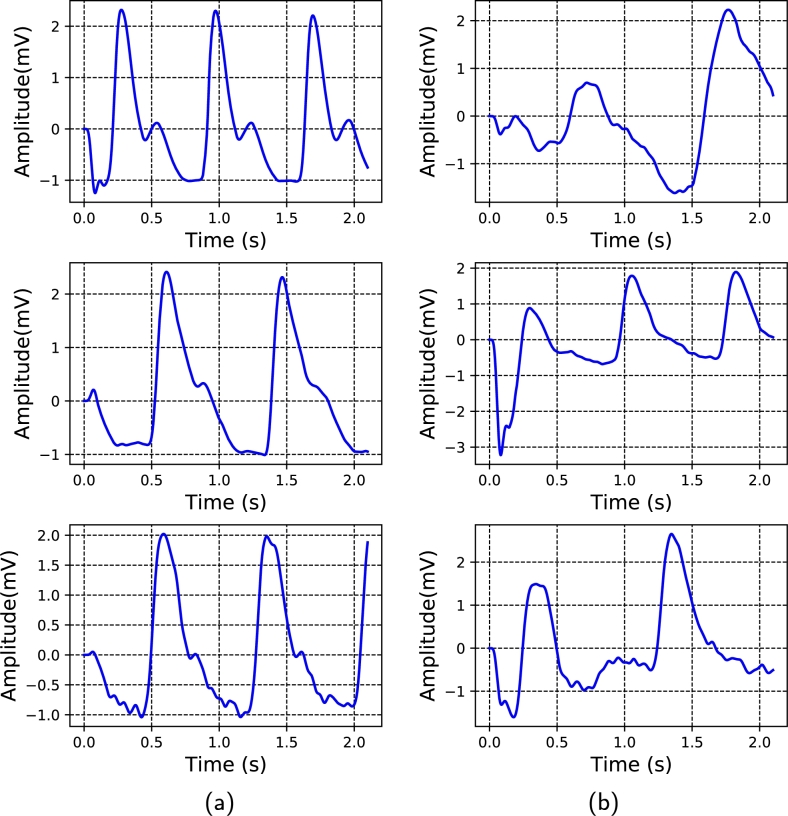
The acceptable (fit) and inappropriate (unfit) PPG waveforms were compared in this study. (a) Appropriate PPG signal (b) Inappropriate PPG signal.

**Figure 4:**
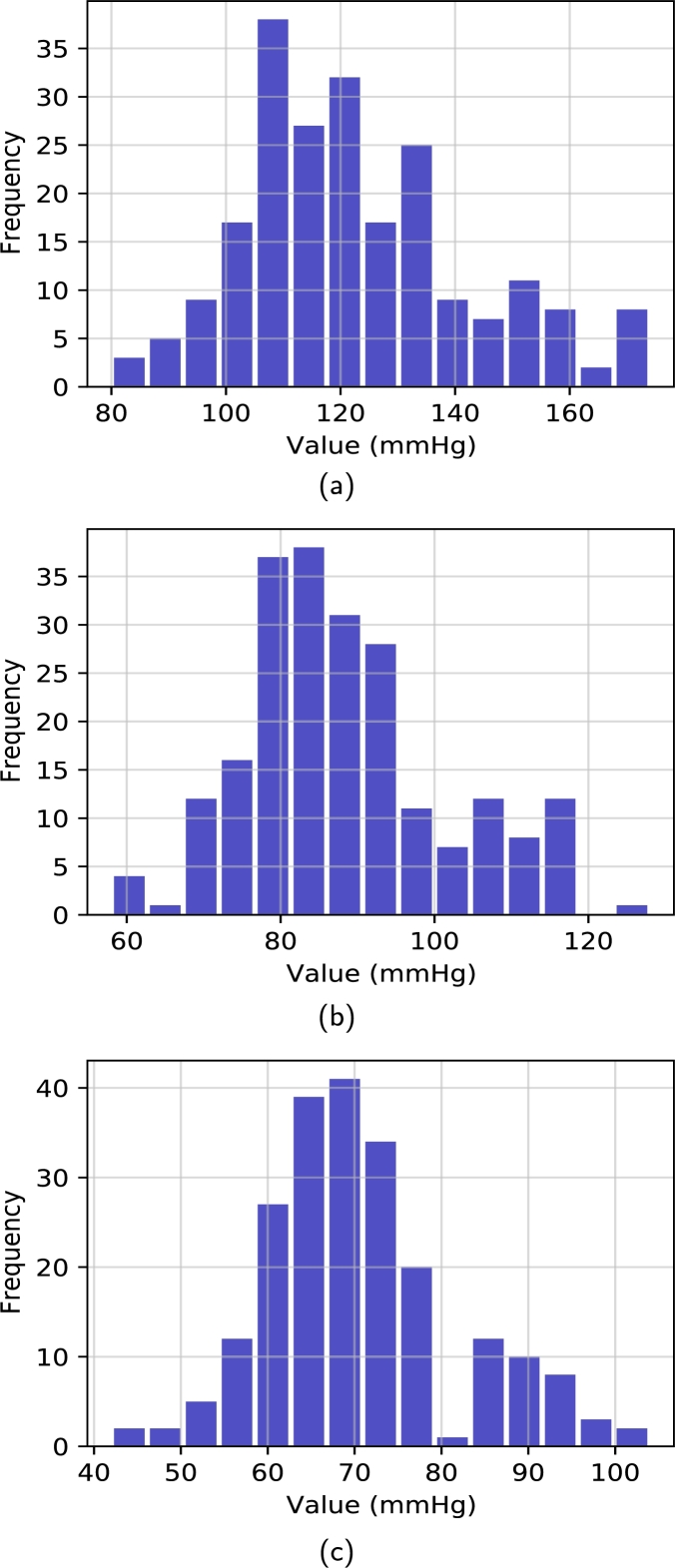
Histograms the database: a) SBP, b) MAP, and c) DBP.

The dataset for this research was taken from Guilin People's Hospital [40], comprising high-quality PPG signals. The authors developed a custom portable hardware system that consisted of a PPG sensor probe, a MSP430FG4618 microcontroller, and a specialized Android application for data management. The PPG sensor was equipped with dual LEDs with 660 nm (red light) and 905 nm (infrared) wavelengths, offering a high sampling rate of 1 kHz, a 12-bit analog-to-digital converter (ADC), and a bandpass hardware filter with a frequency range of 0.5 to 12 Hz. The MSP430FG4618 microcontroller was embedded in the probe to manage the ADC, collect data, and transmit it via Bluetooth to the app. Arterial blood pressure measurements were obtained using the Omron HEM-7201 upper arm monitor. The left index finger was used to collect fingertip PPG signals, and Omron HEM-720 was used to collect reference BP. The nurse assessed BP within the hospital setting. Three PPG segments were collected from each subject during the BP recording. Therefore, the dataset consists of 657 PPG signals collected from 219 subjects, comprising a broad range of SBP and DBP values. The PPG signals were recorded from the left index finger of the subjects with a customized probe. Each signal contained 2100 sampling points with 2.1 s duration, and they were sampled at a rate of 1 kHz. In this study, the collected signals' quality was evaluated using a skewness-based signal quality index (SQI) [41]. In the quality assurance process, 218 signals from 125 subjects were finally kept for this study. To evaluate the skewness quality index, the PPG signal was split into appropriate and inappropriate categories, as illustrated in Fig. 3(a, b). The fit waveforms show visible systolic and diastolic features with dicrotic notches, but the unfit waveforms lack dicrotic notches and other relevant features. Table 1 summarizes the statistical information of the dataset, and Fig. 4 (a − c) demonstrates the histogram of SBP, MAP, and DBP values.

**Table 1:** Patient demographics and clinical laboratory data.

Physical Index	Statistical Data
Age (years)	57 ± 15
Gender	104 male (48%)
Height (cm)	161 ± 8
Weight (kg)	60 ± 11
Systolic Blood Pressure (mmHg)	127 ± 20
Mean Arterial Pressure (mmHg)	87 ± 13
Diastolic Blood Pressure (mmHg)	71 ± 11

* Note: For quantitative variables, the results are expressed as Mean ± S.D. (range) and for categorical variables, as frequency distribution (%).

### 3.2 Preprocessing signals

The raw PPG signals may contain noises like power frequency interference, motion artifacts, and baseline drift [42]. PPG signal preprocessing can be used to reduce noise and improve the quality of the features needed for the feature selection method.

#### 3.2.1 Normalization

**Figure 5:**
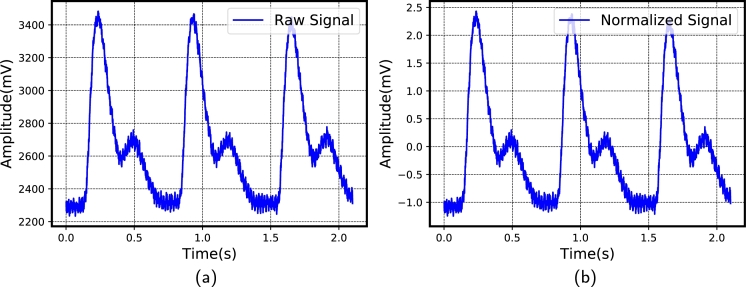
PPG signal. (a) Before normalization (b) After normalization.

Initially, the raw PPG signals are normalized in order to obtain meaningful information from them. In this study, the normalization of the signal is attained using the following equation (2).

(2) $$PPG_{n}=\frac{PPG_{o}-min(PPG_{o})}{max(PPG_{o})-min(PPG_{o})}$$

where $PPG_{o}$ represents the raw PPG signal and $PPG_{n}$ indicates the normalized PPG signal. After normalizing, other preprocessing procedures were also found to be easier to implement. Fig. 5(a, b) shows the sample PPG signal before and after normalization.

#### 3.2.2 Signal filtration

**Figure 6:**
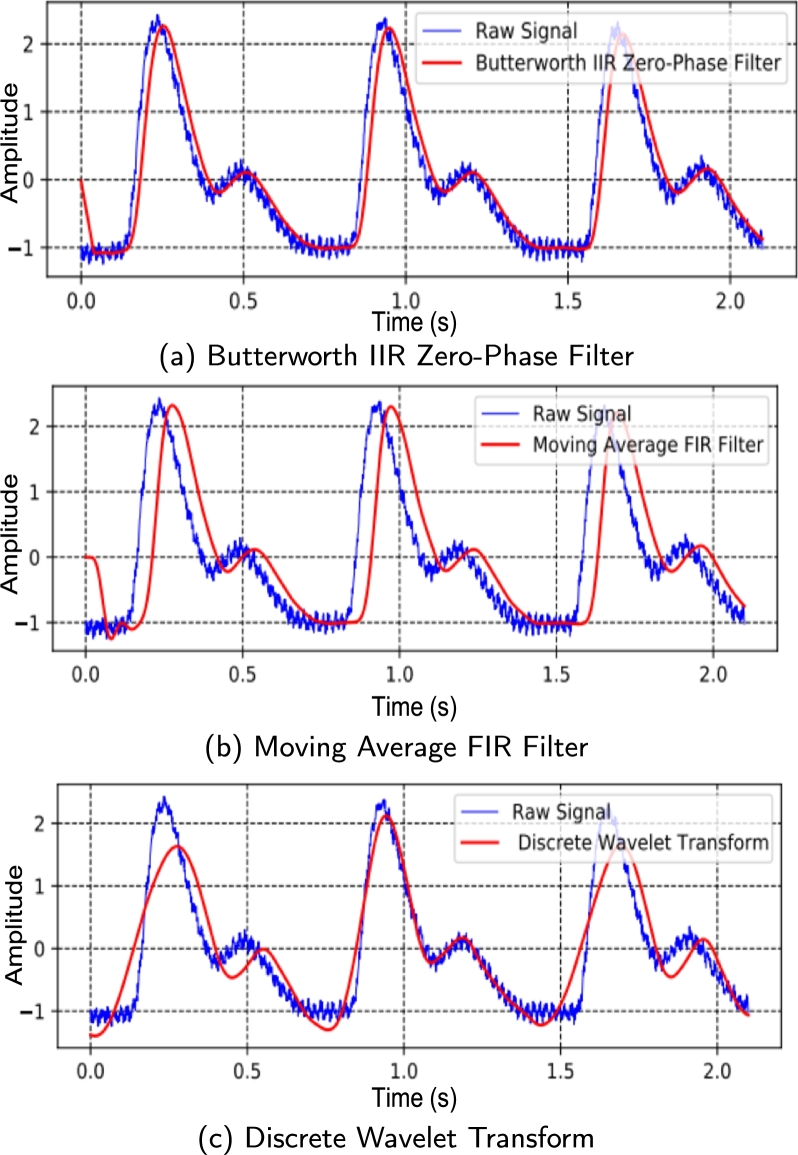
Filtered signals overlaid over the raw PPG signal using various filtering techniques.

The PPG signals from the database [40] was found to have high-frequency noise components. As a result, the signals were filtered using a low-pass filter to eliminate the high-frequency components. Different filtration techniques were examined, such as, $7^{\text{th}}$ order Low Pass Butterworth Filter [43], Moving Average, FIR, and DWT [44]. Fig. 6(a − c) illustrates the raw signal overlaid with the filtered output from several types of filters. From Fig. 6(a), it is shown that Butterworth filter reduces high-frequency noises to obtain more prominent systolic and diastolic peaks required for feature extraction.

### 3.3 Feature extraction

**Figure 7:**
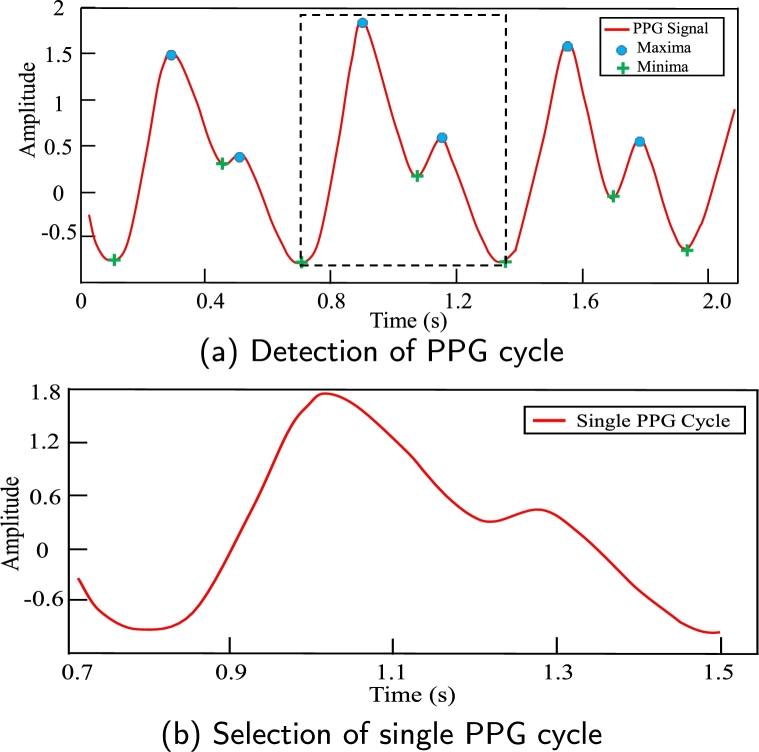
Detection and selection of one single PPG cycle from continuous waveform of PPG signal.

The PPG signal reflects the wave-like blood flow in the vessel from the heart to the fingers and toes [45]. So, the characteristic features of PPG can provide the appropriate information about arterial pressure. As a result, it is vital to find appropriate preprocessing and feature extraction methods for analyzing the PPG signal more accurately [46]. The PPG signal is continuous and repetitive, and each PPG cycle includes about the same amount of information. The signal is then divided into single-period PPG cycles. Although the waveform of single-period PPG cycles may change somewhat from person to person, they are essentially the same in characteristic features [47]. This study used one PPG cycle to extract the characteristic features. A single cycle PPG signal was taken from the 2.1 s time_frame PPG signal to represent a single heartbeat as the 2.1 s time_frame PPG signal contains two or more single cycles. The single PPG cycle with the maximum systolic amplitude was considered the best cycle. The best PPG cycle $PPG_{B}$ was detected and selected automatically using the peak detection algorithm in Algorithm 1. Fig. 7(a, b) depicts the detection of cycle $PPG_{C}$ and the selection of $PPG_{B}$ in $PPG_{S}$.

**Algorithm 1:**
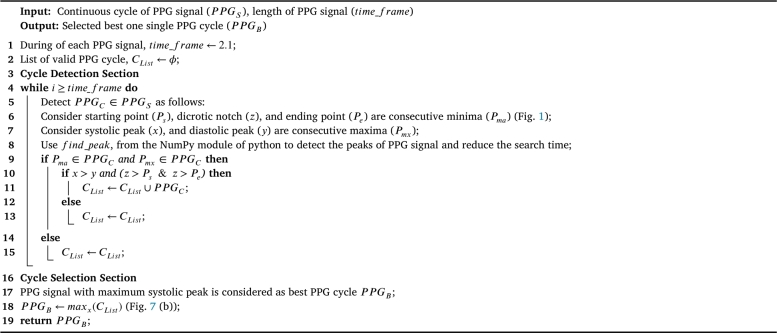
PPG cycle detection and selection algorithm.

**Figure 8:**
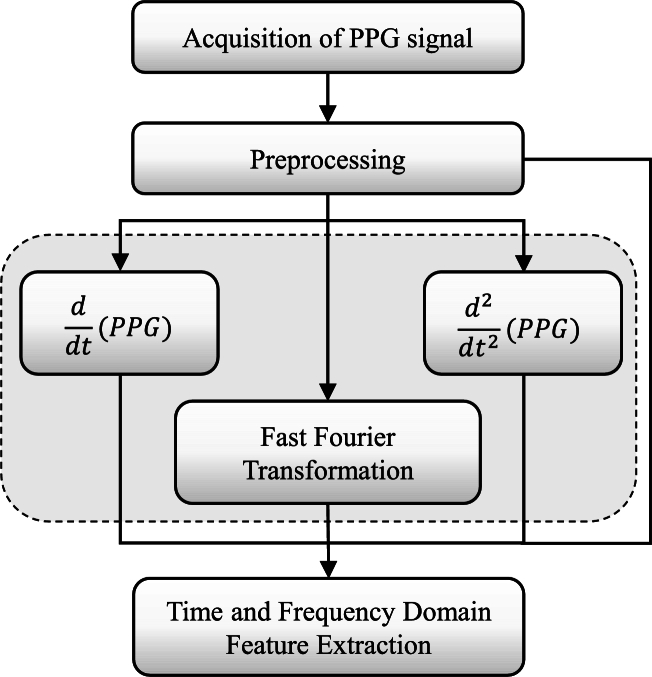
Block diagram of feature extraction.

**Figure 9:**
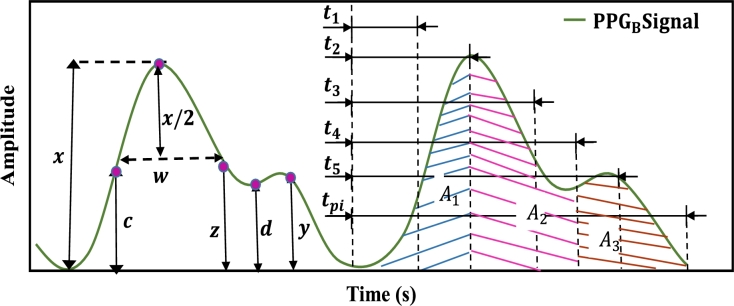
Illustration of time-domain features in a PPG signal.

**Figure 10:**
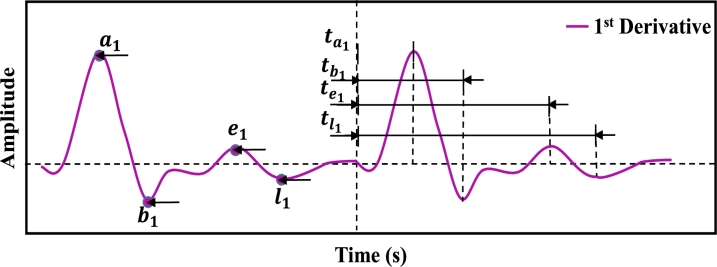
Illustration of time-domain features from first derivatives of PPG signal.

**Figure 11:**
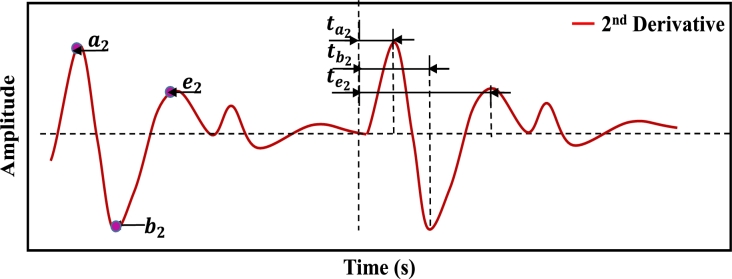
Illustration of time-domain features from 2nd derivatives of PPG signal.

**Figure 12:**
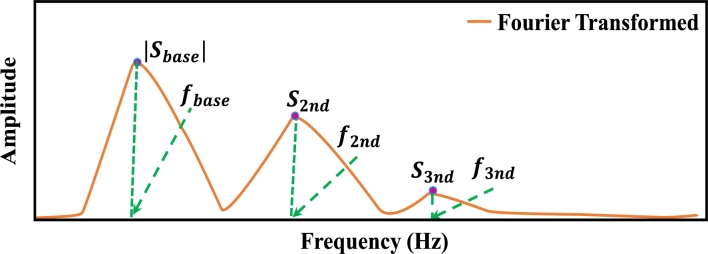
Frequency-domain representation of the PPG signal with its features.

After denoising the PPG signal, 46 features were extracted from $PPG_{B}$ and its derivative signals. The distribution of these characteristic features: the PPG signal yields 21 (1 to 21) features, $1^{\text{st}}$ and $2^{\text{nd}}$ derivatives yield 19 (22 to 40) features, and Fast Fourier Transformation yields 6 (41 to 46) features. In addition, demographic data such as age (47) and gender (48) of each subject were also included as features. Fig. 8 illustrates the block diagram of the feature extraction process. Features with characteristic points of PPG signals are illustrated in Fig. 9. Features extracted from $1^{\text{st}}$ and $2^{\text{nd}}$ derivatives of PPG signal are shown in Figure 10, Figure 11, respectively. The frequency domain feature of the PPG signal is shown in Fig. 12. The details of the extracted features are shown in Table 2.

**Table 2:** Details of features extracted from *PPG*_*B*_ signal, its derivatives and Fourier transform.

Features	Definition	Features	Definition
**PPG signal** − **21 features**			
1. *x*	Systolic peak	2. *y*	Diastolic peak
3. *z*	Dicrotic notch	4. $t_{p_{i}}$	Pulse interval
5. *y*/*x*	Augmentation index	6. (*x* − *y*)/*x*	Alternative augmentation index
7. *z*/*x*	Ratio of dicrotic notch and systolic peak	8. (*y* − *x*)/*x*	Negative relative augmentation index
9. *t*_1_	Systolic peak time	10. *t*_2_	Dicrotic notch time
11. *t*_3_	Diastolic peak time	12. Δ*T* = *t*_3_ - *t*_1_	Systolic and diastolic peaks time difference
13. *w*	Full width at half systolic peak	14. *A*_3_/(*A*_1_ + *A*_2_)	Inflection point area ratio (*IPA*)
15. (*A*_2_ + *A*_3_)/*A*_1_	Ratio of the area before and after dicrotic notch (*sVRI*)	16. *t*_1_/*x*	Systolic peak rising slope
17. *y*/(*t*_*pi*_ − *t*_3_)	Diastolic peak falling slope	18. *t*_1_/*t*_*pi*_	*t*_1_ to *t*_*pi*_ ratio
19. *t*_2_/*t*_*pi*_	*t*_2_ to *t*_*pi*_ ratio	20. *t*_3_/*t*_*pi*_	*t*_3_ to *t*_*pi*_ ratio
21. Δ*T*/*t*_*pi*_	Δ*T* to *t*_*pi*_ ratio		
**PPG 1st derivative (**${\text{PPG}}^{\prime }$**)** − **8 features**			
22. $t_{a_{1}}$	Elapsed time between beginning and peak(*a*_1_)	23. $t_{b_{1}}$	Elapsed time between *a*_1_ and *b*_1_
24. $t_{e_{1}}$	Interval time from point *l*_1_ to point *e*_1_	25. $t_{l_{1}}$	Time interval from point *l*_1_ to next point *l*_1_
26. $t_{a_{1}}/t_{pi}$	Ratio of peak time of PPG′ to *t*_*pi*_	27. $t_{b_{1}}/t_{pi}$	Ratio of valley time of PPG′ to *t*_*pi*_
28. $t_{e_{1}}$/$t_{p_{i}}$	Ratio between time interval of *e*_1_ ($t_{e_{1}}$) and pulse interval ($t_{p_{i}}$)	29. $t_{l_{1}}$/$t_{p_{i}}$	Ratio between time interval of *l*_1_ ($t_{l_{1}}$)
**PPG 2nd derivative (**${\text{PPG}}^{\prime \prime }$**)** − **11 features**			
30. *b*_2_/*a*_2_	Ratio of *b*_2_ and *a*_2_	31. *e*_2_/*a*_2_	Ratio of *e*_2_ and *a*_2_
32. (*b*_2_ + *e*_2_)/*a*_2_	Ratio of (*b*_2_ + *e*_2_) and *a*_2_	33. $t_{a_{2}}$	Time interval from point *l*_2_ to next point *a*_2_ of 2^nd^ derivative PPG
34. $t_{b_{2}}$	Interval time from point *l*_2_ to next point *b*_2_	35. $t_{a_{2}}$/$t_{p_{i}}$	*a*_2_ ($t_{a_{2}}$) to $t_{p_{i}}$ ratio
36. $t_{b_{2}}$/$t_{p_{i}}$	*b*_2_ ($t_{b_{2}}$) to $t_{p_{i}}$ ratio	37. $(t_{a_{1}}+t_{a_{2}})/t_{p_{i}}$	Ratio of $\left(t_{a_{1}}+t_{a_{2}}\right)$ and pulse interval ($t_{p_{i}}$)
38. $(t_{b_{1}}+t_{b_{2}})/t_{p_{i}}$	Ratio of $\left(t_{b_{1}}+t_{b_{2}}\right)$ and pulse interval ($t_{p_{i}}$)	39. $(t_{e_{1}}+t_{2})/t_{p_{i}}$	Ratio of $\left(t_{e_{1}}+t_{2}\right)$ and pulse interval ($t_{p_{i}}$)
40. $(t_{l_{1}}+t_{3})/t_{p_{i}}$	Ratio of $\left(t_{l_{1}}+t_{3}\right)$ and pulse interval ($t_{p_{i}}$)		
**Frequency-domain PPG** − **6 features**			
41. *f*_*base*_	Primary component frequency	42. |*s*_*base*_|	Primary component magnitude
43. $f_{2^{\text{nd}}}$	2^nd^ component frequency	44. $\left|s_{2^{\text{nd}}}\right|$	2^nd^ component magnitude
45. $f_{3^{\text{rd}}}$	3^nd^ component frequency	46. $\left|s_{3^{\text{rd}}}\right|$	2^nd^ component magnitude

### 3.4 Feature selection

Feature selection is the process of choosing appropriate features that enhance the effectiveness and performance of a model [48]. There are various advantages of feature selection prior to model development. Firstly, it eliminates the possibility of overfitting by removing unnecessary features. Second, this process eliminates redundant features, reducing the likelihood of misinterpretation and increasing model accuracy. Finally, it decreases the number of features, simplifying the algorithm and speeding up model training. There are several feature selection methods to select the optimal feature set. In this study, CFS and ReliefF methods are applied.

#### 3.4.1 CFS

Correlation is a statistical term that indicates a feature's relevance with other features. Generally, a feature would be considered good if it has relationships with the class/output but is not redundant. It is measured using a correlation coefficient $R_{cfs}$ or *p*−value.

A correlation-based fitness function is calculated to evaluate fitness in our study. Let the *n*−dimensional feature vectors are $X_{1}$, $X_{2}$, ⋯, $X_{n}$ and the corresponding response values are $y_{1}$, $y_{2}$, ⋯, $y_{n}$. The distance for each pair {$y_{i}$, $y_{j}$} of response values can be expressed as equation (3).

(3) $$E_{y}=y_{i}-y_{j}$$

At the same time, the distance of the corresponding feature values can be expressed as equation (4).

(4) $$E_{X}=\left\{ \begin{array}{cc} \sqrt{\frac{\sum_{k=1}^{n}{\left(X_{i,k}-X_{j,k}\right)}^{2}}{n}}, & \text{if}\;E_{y}\geq 0\\ -\sqrt{\frac{\sum_{k=1}^{n}{\left(X_{i,k}-X_{j,k}\right)}^{2}}{n}}, & \text{if }E_{y}<0 \end{array}\right.$$

Finally, the correlation matrix $R_{cfs}$ between $E_{X}$ and $E_{y}$ can be calculated as equation (5).

(5) $$R_{cfs}=\frac{\frac{\sum_{i}(E_{X_{i}}-{\bar{E}}_{X})(E_{y_{i}}-{\bar{E}}_{y})}{n-1}}{\sqrt{\frac{\sum_{i}{\left(E_{X_{i}}-{\bar{E}}_{X}\right)}^{2}}{n-1}\frac{\sum_{i}{\left(E_{y_{i}}-{\bar{E}}_{y}\right)}^{2}}{n-1}}}$$

The goal is to maximize the value of $R_{cfs}$. After applying the CFS to the feature set, the number of features has been reduced from 48 to 16 for MAP and DBP, and 15 for SBP, respectively. Table 3 lists the CFS based selected features.

**Table 3:** Feature selected by CFS based feature selection algorithm.

Algorithms	SBP	MAP	DBP
CFS	48. Age	48. Age	48. Age
	47.Gender	47. Gende	47. Gender
	45. $f_{3^{\text{rd}}}$	45. $f_{3^{\text{rd}}}$	45. $f_{3^{\text{rd}}}$
	43. $f_{2^{\text{nd}}}$	44. $\left|s_{2^{\text{nd}}}\right|$	44. $\left|s_{2^{\text{nd}}}\right|$
	41. *f*_*base*_	41. *f*_*base*_	42. |*s*_*base*_|
	40. $(t_{l_{1}}+t_{3})/t_{p_{i}}$	40. $(t_{l_{1}}+t_{3})/t_{p_{i}}$	40. $(t_{l_{1}}+t_{3})/t_{p_{i}}$
	29. $t_{l_{1}}$/$t_{p_{i}}$	29. $t_{l_{1}}$/$t_{p_{i}}$	29. $t_{l_{1}}$/$t_{p_{i}}$
	28. $t_{e_{1}}$/$t_{p_{i}}$	28. $t_{e_{1}}$/$t_{p_{i}}$	28. $t_{e_{1}}$/$t_{p_{i}}$
	25. $t_{l_{1}}$	25. $t_{l_{1}}$	26. $t_{a_{1}}$/$t_{p_{i}}$
	14. *A*_2_/*A*_1_	20. *t*_3_/*t*_*pi*_	25. $t_{l_{1}}$
	12. Δ*T* = *t*_3_ − *t*_1_	14. *A*_2_/*A*_1_	14. *A*_2_/*A*_1_
	6. (*x* − *y*)/*x*	12. Δ*T* = *t*_3_ − *t*_1_	12. Δ*T* = *t*_3_ − *t*_1_
	4. *t*_*pi*_	8. (*y* − *x*)/*x*	7. *z*/*x*
	3. *z*	6. (*x* − *y*)/*x*	6. (*x* − *y*)/*x*
	2. *y*	4. *t*_*pi*_	4. *t*_*pi*_
	–	2. *y*	3. *z*

#### 3.4.2 ReliefF

ReliefF uses statistical approaches to choose important characteristics and does not rely on heuristic structure [49]. It takes linear time to handle the number of given features and training samples. It has a structure that applies to both continuous and binary data. However, in terms of fewer training samples and unnecessary features, it cannot make accurate distinctions.

Let training instances sets be $I_{1}$, $I_{2}$, ⋯, $I_{N}$, where *N* is the number of training instances and the feature sets be $F_{1}$, $F_{2}$, ⋯, $F_{n}$. The number of random training instances is *m* out of *N*. ReliefF calculates a proxy statistic for each feature, referred to as feature weights ($W_{F_{i}}$ = weight of feature $F_{i}$, where 1 ≤i≤n). All feature weights are initialized to zero as in equation (6).

(6) $$W_{F_{i}}(t)=0.0$$

The ReliefF algorithm cycles through *m* random training instances (1 ≤t≤m) for updating the feature weight based on feature value differences observed between the target and neighbouring instances. The feature weights are updated as equation (7):

(7) $$W_{F_{i}}(t)=W_{F_{i}}(t-1)-\frac{\Delta F_{i}(R_{t},H)}{m}+\frac{\Delta F_{i}(R_{t},M)}{m}$$

where $R_{t}$ = randomly selected ‘target’ instance, *H* = nearest hit, *M* = nearest miss, and the difference in feature values is calculated as equation (8).

(8) $$\Delta F_{i}(I_{1},I_{2})=\left\{ \begin{array}{cc} 0, & \text{if value }(F_{i},I_{1})\text{ = Value }(F_{i},I_{2})\\ 1, & \text{otherwise} \end{array}\right.$$

After applying the ReliefF to the feature set, the number of features has been reduced from 48 to 15 for SBP and DBP and 16 for MAP, respectively. Table 4 lists the ReliefF-based selected features.

**Table 4:** Feature selected by ReliefF feature selection algorithms.

Algorithms	SBP	MAP	DBP
ReliefF	48. Age	48. Age	48. Age
	47. Gender	47. Gender	47. Gender
	44. $\left|s_{2^{\text{nd}}}\right|$	39. $(t_{e_{1}}+t_{2})/t_{p_{i}}$	45. $\left|f_{3^{\text{nd}}}\right|$
	42. |*s*_*base*_|	38. $(t_{b_{1}}+t_{b_{2}})/t_{p_{i}}$	42. |*s*_*base*_|
	28. $t_{e_{1}}/t_{pi}$	37. $(t_{a_{1}}+t_{a_{2}})/t_{p_{i}}$	31. *e*_2_/*a*_2_
	27. $t_{b_{1}}/t_{pi}$	36. $t_{b_{2}}$/$t_{p_{i}}$	26. $t_{a_{1}}/t_{pi}$
	25. $t_{l_{1}}$	35. $t_{a_{2}}$/$t_{p_{i}}$	25. $t_{l_{1}}$
	16. *t*_1_/*x*	34. $t_{b_{2}}$	16. *t*_1_/*x*
	14. *A*_2_/*A*_1_	31. *e*_2_/*a*_2_	12. Δ*T* = *t*_3_ − *t*_1_
	8. (*y* − *x*)/*x*	29. $t_{l_{1}}$/$t_{p_{i}}$	8. (*y* − *x*)/*x*
	7. *z*/*x*	28. $t_{e_{1}}$/$t_{p_{i}}$	7. *z*/*x*
	6. (*x* − *y*)/*x*	24. $t_{e_{1}}$	6. (*x* − *y*)/*x*
	5. *y*/*x*	18. *t*_1_/*t*_*pi*_	5. *y*/*x*
	3. *z*	12. Δ*T* = *t*_3_ - *t*_1_	3. *z*
	2. *y*	10. *t*_2_	2. *y*
	–	4. $t_{p_{i}}$	–

### 3.5 Model construction and validation

This paper implemented four nonlinear regression methods to construct the BP estimation model based on all the features and features selected by the CFS and ReliefF algorithms. The linear regression algorithm cannot be explained due to the nonlinear relationship between PPG signals and BP values. Moreover, the findings of the linear regression method applied to the database of this paper do not yield acceptable results according to the standards of the Advancement of Medical Instrumentation standard (AAMI) [50] and the British Hypertension Society standard (BHS) [51]. The 10-fold cross-validation approach splits the training and validation sets to obtain the optimal prediction model. To avoid the overfitting and underfitting of the models and improve the predictive performance of the models, this paper used a grid search algorithm to determine the optimal hyperparameter of each model. In addition, the used parameters and their associated values used in machine learning algorithms are enlisted in Table 5.

**Table 5:** The parameters and their associated values that are used in machine learning algorithms.

Models	Parameters	Settings (values)
SVR	regularization *C*	100
	margin tolerance *ϵ*	0.1
	kernel	RBF
RFR	n_estimator	150
	criterion	mae
	max depth	13
DTR	criterion	mae
	min samples leaf	17
	max depth	12
	min samples splits	5
KNR	number of neighbours	51
	leaf size	45
	distance	Euclidean

#### 3.5.1 Support vector regression (SVR)

Support vector machine (maximum-margin) might be utilized as a regression method by using kernels such as radial basis function (RBF) to reflect nonlinear relationships [52]. In support vector regression, the input feature set is first mapped to a high dimensional space through nonlinear mapping and establishes the optimal hyperplane. Suppose the input feature set is *F*= {$f_{1}$, $f_{2}$, ⋯, $f_{n}$} and the corresponding reference value of blood pressure is $y_{1}$, $y_{2}$, ⋯, $y_{n}$. The linear model constructed for BP estimation in this study is expressed as equation (9).

(9) $$BP_{e}=\sum_{i=1}^{m}\omega_{i}\varphi_{i}(F)+b$$

where $\varphi_{i}(F)$ denotes a set of nonlinear transformations, $\omega_{i}$ is the coefficient of the regression function, and *b* is the biasing value.

#### 3.5.2 Random forest regression (RFR)

The random forest is an ensemble model operated by forming several decision trees during training time [53], [54]. When the final assessed value of a RF is continuous, the method is called RFR. The RFR model is constructed in this study by taking the most relevant features as the input parameters, and the out-of-package data set is divided by the self-sampling method. The model continuously divides the optimal variables according to the values of entropy and variance and forms several decision trees. The final BP is estimated by averaging each decision tree's outcomes. The mathematical model for BP estimation can be indicated as equation (10):

(10) $$BP_{e}=\frac{1}{B}\sum_{b=1}^{B}T_{b}(x)$$

where $T_{b}$ denotes a set of regression trees, and $T_{b}(x)$ is the estimation value of $T_{b}$. In addition, the model has a high capacity for generalization, can tolerate a limited number of outliers and missing variables, and has a fast training speed.

#### 3.5.3 Decision tree regression (DTR)

The decision tree can also be applied to both classification and regression situations. The model is called decision tree regression when a decision tree estimates a continuous value. In our study, a decision tree is formed top-down from a root node and splits the dataset into subsets as nodes that reflect values for the characteristic investigated. Determining the attribute of the data to the decision tree is the most significant aspect that leads to a more excellent resolution. After that, the features are ranked to generate a decision for BP estimation [55].

#### 3.5.4 K-nearest neighbor regression (KNR)

KNR is a non-parametric strategy that averages the numerical target of the K-nearest neighbours and approximates the association between independent variables and the continuous outcome [56]. The KNR makes predictions based on results from the neighbour *K* closest to that point. For estimating BP with KNR, firstly, we calculate the distance between the request point and the case reference point from the dataset. As the Euclidean distance is only applicable for continuous variables like PPG signals, we use it to calculate distance as in equation (11).

(11) $$E_{d}=\sqrt{\sum_{i=1}^{K}{\left(x_{i}-y_{i}\right)}^{2}}$$

where $E_{d}$ stands for Euclidean distance, with $x_{i}$ denoting the query point and $y_{i}$ denoting a case from the dataset sample. Then we select the value of *K* using trial and error to estimate blood pressure. For KNR, prediction is the average of the K-nearest neighbors' outcome as in equation (12).

(12) $$BP_{e}=\frac{1}{K}\sum_{i=1}^{K}y_{i}$$

where $BP_{e}$ stands for estimated BP, and $y_{i}$ stands for the $i^{\text{th}}$ case of the examples sample.

## 4 Experimental result and discussion

### 4.1 Evaluation criteria

The coefficient of determination ($R^{2}$), mean absolute error (MAE), root mean square error (RMSE), mean square error (MSE), mean difference (ME), and standard deviation (STD) have been utilized as the performance measurement criteria to evaluate our proposed BP models. The mathematical formulas for these indices are indicated as in equations (13)–(18).

(13) $$R^{2}=1-\frac{\sum_{n}{\left(BP_{a}-BP_{e}\right)}^{2}}{\sum_{n}{\left(BP_{a}-\bar{BP}\right)}^{2}}$$

(14) $$MAE=\frac{1}{n}\sum_{n}\left|BP_{a}-BP_{e}\right|$$

(15) $$MSE=\frac{1}{n}\sum_{n}{\left(BP_{a}-BP_{e}\right)}^{2}$$

(16) $$RMSE=\sqrt{MSE}$$

(17) $$ME=\frac{1}{n}\sum_{n}(BP_{a}-BP_{e})$$

(18) $$STD=\sqrt{\frac{\sum_{n}{\left(BP_{a}-BP_{e}-ME\right)}^{2}}{n-1}}$$

where $\bar{BP}=\frac{1}{n}\sum_{n}BP_{a}$ and $BP_{a}$ is the actual or reference value while $BP_{e}$ is estimated value.

### 4.2 Robustness performance of the models

The performance of the nonlinear models has been evaluated with six performance measurement indices: $R^{2}$, MAE, RMSE, MSE, ME and STD. It is noteworthy that the parameter $R^{2}$ defines the relationship of the estimated value with the reference value. The closer $R^{2}$ is to 1, the stronger the link between model outputs and reference values. The RMSE and MSE elucidate relative errors, MAE measures absolute errors, ME measures the estimation of BP's bias, and STD is usually used to measure the error variability.

There were 219 patients in this study, ranging in age from 21 to 85 years old, with 104 men and 115 females. For this group, the actual or reference SBP varied from 80 to 174 mmHg, with SBP mean values of 127 mmHg and a standard deviation of 20 mmHg. The actual or reference MAP range ranged from 58 mmHg to 128 mmHg for this individual sample, with mean MAP values of 87 mmHg and standard deviation of MAP of 13 mmHg. Similarly, for the DBP sample, the reference or measured DBP ranged between 42 mmHg and 104 mmHg, with a mean of 71 mmHg and a standard deviation of DBP of 11 mmHg.

As noted, extracted feature vectors are fed into nonlinear regression algorithms (SVR, RFR, DTR, and KNR) as inputs. Each nonlinear regression model estimates each BP value (DBP, MAP, and SBP) by separating training and testing data. In the first stage, SVR, RFR, DTR, and KNR are trained and validated with all features for BP estimation. We split our dataset into training (80%) and test (20%) sets based on ID. It is also noted that each recorded signal of the PPG-BP Database has its unique ID. Our study used it to prevent overlapping the subjects of the training and testing sets. The 10-fold cross-validation method was applied to validate each model. In 10-fold, each fold contained the reference BP values and corresponding feature vectors. Table 6 demonstrates the performance measurement evaluation of the models with all features. According to the obtained result in Table 6, SVR with all features provides better accuracy of $R^{2}=0.71$ and MAE=11.78 mmHg for SBP, 0.72 and 5.53 mmHg for MAP, 0.61 and 7.89 mmHg for DBP, respectively.

**Table 6:** Performance measurement of BP using various learning algorithms with all features.

	Algorithm	*R*^2^	MAE	RMSE	MSE
SBP	SVR	0.71	11.78	14.85	239.87
	RFR	0.57	14.65	17.89	320.54
	DTR	0.61	12.89	17.85	301.87
	KNR	0.56	14.99	18.78	327.08

MAP	SVR	0.72	5.53	9.72	94.63
	RFR	0.68	6.33	10.57	111.8
	DTR	0.69	6.02	10.27	105.5
	KNR	0.63	6.56	11.14	124.27

DBP	SVR	0.61	7.89	9.78	105.27
	RFR	0.56	8.25	10.85	112.78
	DTR	0.54	8.78	10.47	114.59
	KNR	0.43	9.65	11.30	127.41

In the next stage, a CFS-based feature selection technique is applied to select the best optimal features. Correlation measures whether a feature strongly correlates with a class label but not with any other relevant feature. After applying CFS to the feature set, the number of features is reduced from 48 to 16 for MAP and DBP and 15 for SBP, respectively. The results for nonlinear learning algorithms with CFS have been shown in Table 7. Table 7 shows that the appropriate features vary according to the measurement level. It occurs as a result of the fact that certain features are associated with different BP (SBP, MAP, and DBP) levels. According to the obtained results in Table 7, it can be observed that CFS, along with the SVR model, provides better accuracy of $R^{2}$ = 0.90 and *MAE* = 3.82 for SBP, 0.91 and 2.62 mmHg for MAP, 0.92 and 2.97 mmHg for DBP, respectively. From Table 6, Table 7, it is noticed that the accuracy ($R^{2}$) of the SVR model with CFS-based features selection is increased by 26.76%, 26.38%, and 48.38% respectively for SBP, MAR and DBP levels compared with the SVR model with all features.

**Table 7:** Performance measurement of BP using various learning algorithms with CFS based selected features.

	Algorithm	*R*^2^	MAE	RMSE	MSE
SBP	SVR	0.90	3.82	5.28	28.74
	RFR	0.84	7.62	10.31	106.61
	DTR	0.87	6.58	8.92	80.64
	KNR	0.82	8.32	12.55	159.25

MAP	SVR	0.91	2.62	3.89	15.13
	RFR	0.84	3.80	6.40	40.96
	DTR	0.90	2.91	4.18	17.47
	KNR	0.86	3.42	6.12	37.45

DBP	SVR	0.92	2.97	4.84	23.43
	RFR	0.85	4.21	8.37	70.16
	DTR	0.88	3.45	6.21	38.64
	KNR	0.81	4.97	9.02	81.36

Furthermore, the ReliefF-based feature selection technique is applied to select the best optimal features. The selection of features is required to minimize the possibility of model over-fitting and computational complexity. ReliefF is a feature selection technique that randomly chooses instances and weights them based on their nearest neighbours [49]. After applying ReliefF to the feature set, the number of features has been reduced from 48 to 15 for SBP and DBP and 16 for MAP, respectively. The results for nonlinear learning algorithms with ReliefF have been shown in Table 8. Among CFS and ReliefF, ReliefF selects the most promising features with higher accuracy than the CFS algorithm. According to the obtained results in Table 8, it can be observed that ReliefF, along with the SVR model, provides the best-estimated accuracy of $R^{2}$ = 0.93 and *MAE* = 2.49 for SBP, 0.95 and 1.62 mmHg for MAP, 0.95 and 1.69 mmHg for DBP, respectively. From Table 6, Table 8, it is noticed that the accuracy ($R^{2}$) of the SVR model with ReliefF-based feature selection is increased by 30.98%, 31.94%, and 55.73% respectively for SBP, MAR and DBP levels compared with the SVR model with all features.

**Table 8:** Performance measurement of BP using various learning algorithms with ReliefF based selected features.

	Algorithm	*R*^2^	MAE	RMSE	MSE
SBP	**SVR**	**0.93**	**2.49**	**3.45**	**12.78**
	RFR	0.85	7.24	12.60	160.76
	DTR	0.91	3.32	6.54	42.77
	KNR	0.83	7.09	10.20	106.04

MAP	**SVR**	**0.95**	**1.62**	**2.52**	**6.38**
	RFR	0.85	3.52	6.37	40.58
	DTR	0.92	2.26	3.31	10.96
	KNR	0.90	2.89	4.10	16.81

DBP	**SVR**	**0.95**	**1.69**	**3.60**	**12.78**
	RFR	0.89	3.19	6.02	36.24
	DTR	0.90	2.80	5.61	30.14
	KNR	0.86	3.98	7.36	54.17

**Figure 13:**
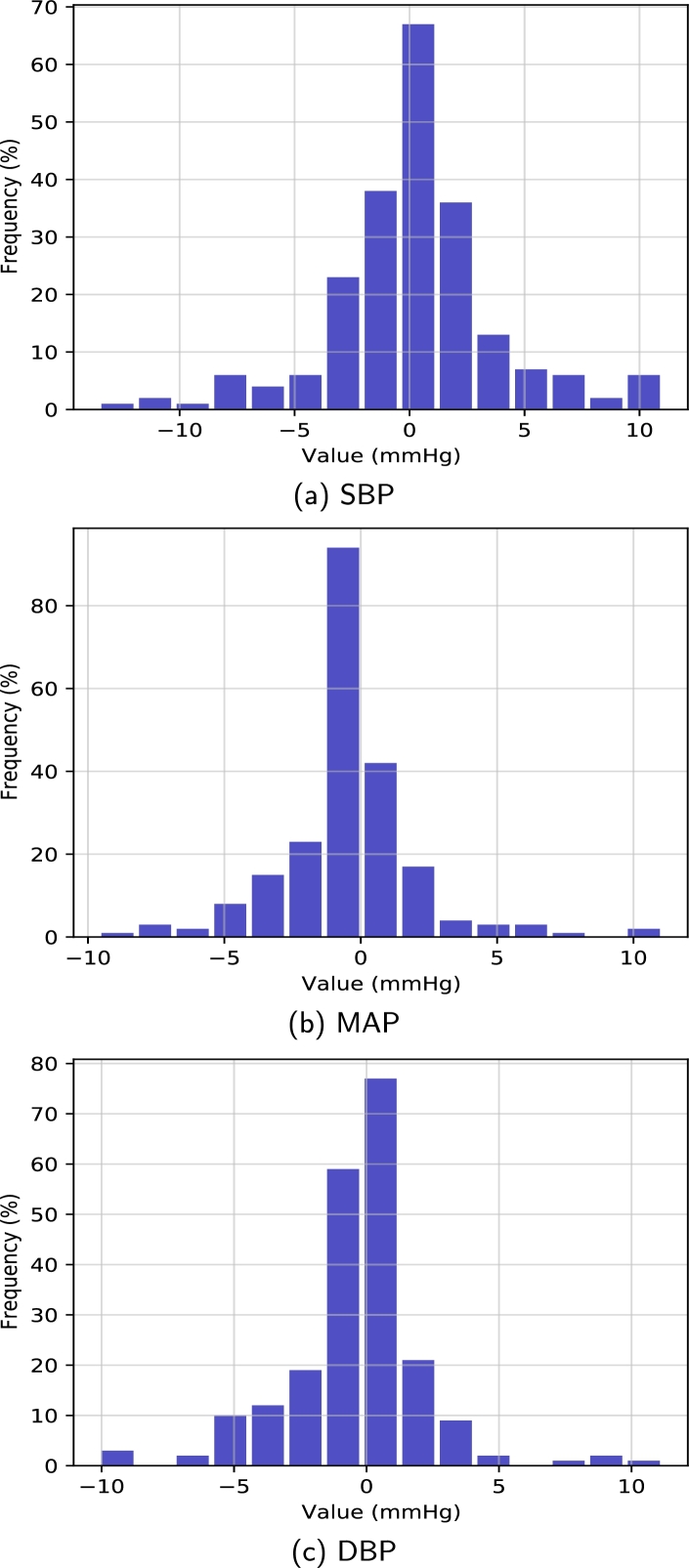
Histograms of estimated errors in SVR based model for BP.

**Figure 14:**
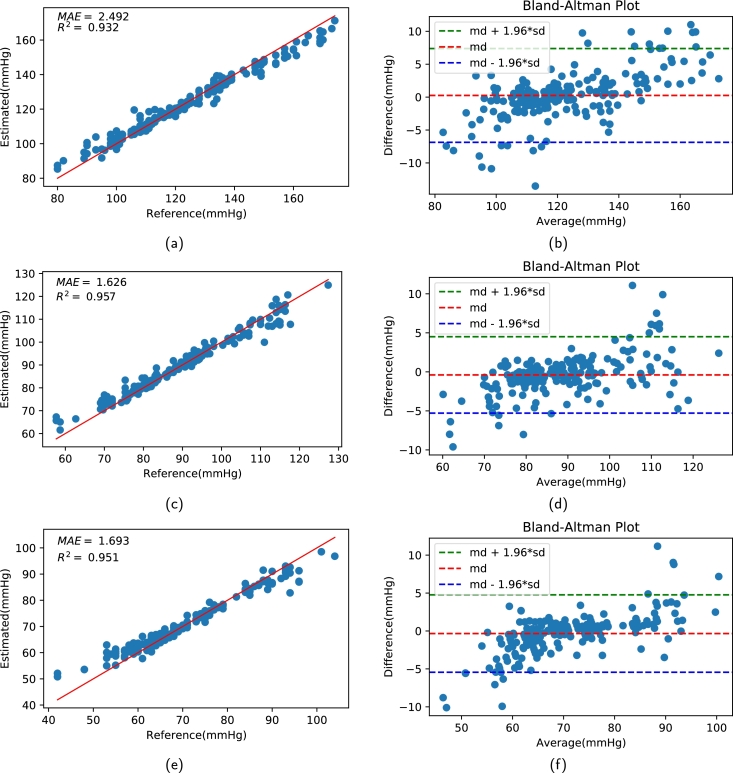
Regression plot and Bland-Altman plot between estimated values and reference value at testing using SVR model with ReliefF based feature selection: Relationship (SBP) (b) Agreement (SBP) (c) Relationship (MAP) (d) Agreement (MAP) (e) Relationship (DBP) (f) Agreement (DBP).

Fig. 13 (a − c) illustrates the histogram of the estimated error in the SVR model and ReliefF for SBP, MAP, and DBP, respectively. The error values for BP estimation in all three histograms are generally distributed around zero. Fig. 14 (a, c, e) shows the regression plots for all BP values. The three linear regression forms illustrate the relationship between the estimated and reference values for SBP, MAP, and DBP. The bland-Altman (B&A) plot describes the agreement between two quantitive measurements by establishing limits of agreement (LOA). In Fig. 14 (b, d, f), the red line depicts the average difference (*md*) between the two measurements (estimated BP value by the proposed method and the BP value obtained by invasive device), and the green and blue lines depict md+1.96⁎sd and md−1.96⁎sd, respectively, where sd stands for standard deviation. From Fig. 14 (b, d, f), it can be seen that a large percentage of the estimated values for all BP values are within the limits of agreement (md±1.96⁎sd). The limits of agreement for SBP with the SVM model along with ReliefF-based features at a 95% confidence interval was [−0.287 mmHg, 6.98 mmHg], for MAP was [−5.31 mmHg, 4.83 mmHg] and for DBP was [−5.16 mmHg, 4.98 mmHg]. However, the estimations for extremely low or extremely high BP values are not as precise as those for other samples. This problem is mainly caused by the small number of subjects with extremely high or extremely low blood pressure in the training dataset, which hinders the predictive performance of the regression model for unusual BP values.

### 4.3 Evaluation using the AAMI standard

Table 9 demonstrates the results of using the ReliefF-based selected features and the SVR model with the AAMI criterion. BP measuring equipment must have ME and STD values of less than 5 and 8 mmHg, respectively, according to the AAMI [57]. Moreover, based on this standard, at least 85 individuals must participate in the experiment to ensure the method's accuracy. According to Table 9, the results of the proposed method for all three BP estimations are fully approved by the maximum acceptable ME and STD values.

**Table 9:** Comparison of this study results with AAMI.

		ME (mmHg)	STD(mmHg)	Subjects
AAMI [57]	BP	≤5	≤8	≥85
Our Results	SBP	0.533	4.175	125
	MAP	−0.299	2.441	125
	DBP	−0.049	2.188	125

### 4.4 Evaluation using the BHS standard

Furthermore, the result of our proposed method is verified by the BHS shown in Table 10. BHS assesses blood pressure measurement devices according to their cumulative percentage of errors with 5, 10 and 15 mmHg thresholds [58]. The proposed algorithms achieved Grade A for all BP estimations per this criterion.

**Table 10:** Comparison of this paper results with BHS.

		Cumulative Error Percentage		
		≤5 mmHg	≤10 mmHg	≤15 mmHg
Our Results	SBP	91%	95%	99%
	MAP	93%	96%	100%
	DBP	94%	96%	100%

BHS [58]	Grade A	60%	85%	95%
	Grade B	50%	75%	90%
	Grade A	40%	65%	85%

### 4.5 Comparison with other works

Table 11 shows previous studies that have used the PPG to estimate BP and tested their methods on various datasets. A meaningful comparison of BP estimation cannot be conducted since these studies employed datasets and measurement criteria for evaluation that are different from the proposed methodology.

**Table 11:** Comparison of our proposed method with several existing cuff-less BP estimation process using PPG signals.

Work	Number of Subjects	Method	Validation Method	Performance		
				(MAE±STD) mmHg		
				SBP	MAP	DBP
Mousavi et al. [12]	MIMIC-II (441 subjects)	FFT, PCA, AdaboostR	10-fold cross-validation	3.97 ± 7.99	2.61 ± 4.16	2.43 ± 3.37

Kachuee et al. [26]	MIMIC-II(3663 records)	DWT, AdaBoost, Random Forest	10-fold cross-validation	11.17 ± 10.09	5.92 ± 5.38	5.35 ± 6.14

Chowdhury et al. [28]	222 recordings, 126 subjects	Feature extraction, statistical, and demographic features, ReliefF, CFS, GPR	10-fold cross-validation	3.02 ± 9.29	—	1.74 ± 5.54

Mousavi et al. [29]	MIMIC II(660 records)	FFT, FFT^−1^, feature extraction, PCA and RFR	10-fold cross-validation	13.04 ± 12.80	7.23 ± 7.14	5.96 ± 5.67

Zhang et al. [30]	The University of Queensland Vital Signs Dataset (32 cases, 7000 samples)	Feature extraction, CFS, SVM	Conventional method	11.64 ± 8.20	—	7.61 ± 6.78

Slapničar et al. [31]	MIMIC-III(510 subjects)	Temporal and frequency features of PPG, PPG′ and PPG″, Spectro-temporal Resnet	Conventional method	6.88	—	9.43

Dey et al. [33]	Own dataset (205 subjects)	Lasso Regression	Conventional method	6.9 ± 9.0	—	5.0 ± 6.1

Liu et al. [35]	MIMIC II (910 records)	Support Vector Regression	Conventional method	8.54 ± 10.9	—	4.34 ± 5.8

Duan et al. [36]	The University of Queensland Vital Signs Dataset (32 cases, 7000 samples)	Wavelet transformation, Support Vector Regression	10-fold cross-validation	4.77 ± 7.68	—	3.67 ± 5.69

El-Hajj et al. [13]	MIMIC II (942 subjects)	DWT, BiLSTM + LSTM + Attention	Conventional method	4.51 ± 7.81	—	2.6 ± 4.41

Hasanzadeh et al. [17]	University of California, Irvine (UCI) repository (1000 subjects)	AMPD, AdaBoostR	10-fold cross-validation	8.22 ± 10.38	—	4.17 ± 4.22

**Our Proposed Method**	218 recordings, 125 subjects	Time & frequency domain features of PPG, PPG′ and PPG″, ReliefF, CFS, SVR	10-fold cross-validation	2.49 ± 7.82	1.62 ± 5.47	1.69 ± 4.02

* FFT = Fast Fourier Transform, ${\text{FFT}}^{-}1$ = Inverse Fast Fourier Transform, PCA = Principal Component Analysis, RFR = Random Forest Regression, VIF = Variance inflation factor, AdaBoostR = Adaptive Boosting regression, AMPD = Automatic Multi-scale-based Peak Detection, MLR = Multiple Linear Regression, SVM = Support Vector Machine, CFS = Correlation-based feature selection, GPR = Gaussian Process Regression, SVR = Support Vector Regression.

Looking at individual previous works in Table 11, the number of samples varied from 222 to 7000. In [29], authors compared four machine learning algorithms and presented that random forest regression achieved the lowest error percentage. Kachuee et al. [26] also tested several regression models: regularized linear regression, decision tree regression, support vector machine, AdaBoost, and random forest regression. Among them, AdaBoost and random forest regression achieved acceptable accuracy in estimating. Several other studies, including this one, also use support vector machines and their extension, support vector regression, in their regression stages [30], [35], [36]. However, the findings varied depending on the dataset, preparation processes, and other variables. Dey et al. [33] proposed a smartphone-based monitoring system. They used a lasso regression model to achieve acceptable accuracy. Slapničar et al. [31] achieved a reasonable accuracy using spectro-temporal Resnet. Zhang et al. [30] and Chowdhury et al. [59] attained a fair accuracy, although in a small dataset. Hasanzadeh et al. [17] and Mousavi et al. [12] employed the AdaBoost regression model and achieved promising results. Chowdhury et al. [28] achieved a good result using Gaussian process regression in the same dataset we have used.

Furthermore, our proposed method obtained an average MAE±STD of 2.49±7.82 mmHg for SBP, 1.62±5.47 mmHg for MAP and 1.43±4.02 mmHg for DBP estimation which are better compared to the existing methods. These findings illustrate the effectiveness of our proposed methodology for real-time clinical application usability.

## 5 Conclusion

In this study, we proposed a non-invasive and cuffless BP estimation method based on characteristic features of PPG signals and nonlinear regression models. While regarded as the more dependable option, cuff-based devices are typically uncomfortable and unsuitable for continuous blood pressure monitoring. This study shows how to reliably estimate blood pressure without relying on cuff-based approaches. The proposed methodology includes signal preprocessing, like the quality assessment of PPG signals and signal filtration, feature extraction, feature selection, and finally, regression model development. Firstly, PPG signals are obtained from 219 subjects. Then, after denoising the PPG signal and its derivatives, 46 characteristic features are extracted. CFS and ReleifF feature selection techniques are applied to select the appropriate features. Finally, four nonlinear regression models are established to estimate BP values, including the SVR, KNR, DTR and RFR models. A 10-fold cross-validation method is applied to validate the models. According to the MAE performance criteria, combining ReleifF-based selected features with the SVR model provides the best accuracy for estimating continuous BP. The BHS and AAMI standards have been used to assess the accuracy of our proposed BP estimation method. The estimated scores met the requirements of the AAMI standard and achieved Grade A for SBP, MAP, and DBP in compliance with the BHS standards.

While promising, the proposed method for cuffless blood pressure estimation has limitations, including small sample size, potential inaccuracies due to PPG signal quality, and reliance on specific feature selection techniques and regression models. It has also yet to be tested in long-term real-world scenarios, and extending to other physiological parameters and smartphone integration presents additional challenges. In the future, we plan to collect data from healthy subjects and expand our work by automatically including the estimation of other physiological parameters, such as blood glucose levels, blood component levels, etc., and also integrate the whole system into a smartphone.

## Ethical approval

Not required.

## CRediT authorship contribution statement

**Araf Nishan:** Writing – original draft, Methodology, Conceptualization. **S. M. Taslim Uddin Raju:** Writing – review & editing, Methodology, Conceptualization. **Md Imran Hossain:** Visualization, Investigation. **Safin Ahmed Dipto:** Writing – original draft, Visualization, Formal analysis. **S. M. Tanvir Uddin:** Writing – review & editing, Project administration. **Asif Sijan:** Validation, Investigation. **Md Abu Shahid Chowdhury:** Writing – review & editing. **Ashfaq Ahmad:** Writing – original draft, Visualization. **Md Mahamudul Hasan Khan:** Supervision.

## Declaration of Competing Interest

The authors declare the following financial interests/personal relationships which may be considered as potential competing interests:

S. M. Taslim Uddin Raju reports was provided by Khulna University of Engineering and Technology. If there are other authors, they declare that they have no known competing financial interests or personal relationships that could have appeared to influence the work reported in this paper.

## Data Availability

The dataset is publicly available at the link: https://www.kaggle.com/datasets/raju32742/dataset-bp-45feature/data.
